# Supplementary material for: COZOID: contact zone identifier for visual analysis of protein-protein interactions
Source: BMC Bioinformatics. 2018 Apr 6;19:125. doi: 10.1186/s12859-018-2113-6 (PMC5889581; doi:10.1186/s12859-018-2113-6)
Supplement: Supplementary file 2 — Software build. Executable binary file of the software tool. (ZIP 118,784 kb) [file 12859_2018_2113_MOESM2_ESM.zip › cozoid/platform/docs/testng-6.5.1-javadoc.zip!/org/testng/annotations/package-summary.html]

org.testng.annotations (TestNG)


---


|  |  |  |
| --- | --- | --- |
| **Overview** \| **Package** \| Class \| **Tree** \| **Deprecated** \| **Index** \| **Help** | |  |
| **PREV PACKAGE**   **NEXT PACKAGE** | **FRAMES**    **NO FRAMES**     **All Classes** |  |


---

## Package org.testng.annotations

| **Interface Summary** | |
| --- | --- |
| **IAnnotation** | The parent interface for all the annotations. |
| **IConfigurationAnnotation** | Encapsulate the @Configuration / @testng.configuration annotation Created on Dec 20, 2005 |
| **IDataProviderAnnotation** | Encapsulate the @DataProvider / @testng.data-provider annotation Created on Dec 20, 2005 |
| **IExpectedExceptionsAnnotation** | Encapsulate the @ExpectedExceptions / @testng.expected-exceptions annotation Created on Dec 20, 2005 |
| **IFactoryAnnotation** | Encapsulate the @Factory / @testng.factory annotation Created on Dec 20, 2005 |
| **IObjectFactoryAnnotation** |  |
| **IParameterizable** | Parent interface for annotations that can receive parameters. |
| **IParametersAnnotation** | Encapsulate the @Parameters / @testng.parameters annotation Created on Dec 20, 2005 |
| **ITestAnnotation** | Encapsulate the @Test / @testng.test annotation. |
| **ITestOrConfiguration** | This interface captures methods common to @Test and @Configuration Created on Dec 20, 2005 |

| **Annotation Types Summary** | |
| --- | --- |
| **AfterClass** |  |
| **AfterGroups** |  |
| **AfterMethod** |  |
| **AfterSuite** |  |
| **AfterTest** |  |
| **BeforeClass** |  |
| **BeforeGroups** |  |
| **BeforeMethod** |  |
| **BeforeSuite** |  |
| **BeforeTest** |  |
| **Configuration** | **Deprecated.** *Use @BeforeSuite, @AfterSuite, @BeforeTest, @AfterTest, \@BeforeGroups, @AfterGroups, @BeforeClass, @AfterClass, @BeforeMethod, \@AfterMethod* |
| **DataProvider** | Mark a method as supplying data for a test method. |
| **ExpectedExceptions** | **Deprecated.** *Use @Test(expectedExceptions = "...")* |
| **Factory** | Marks a method as a factory that returns objects that will be used by TestNG as Test classes. |
| **Guice** | This annotation specifies what Guice modules should be used to instantiate this test class. |
| **Listeners** | This annotation lets you define listeners directly on a test class instead of doing so in your testng.xml. |
| **NoInjection** | Turn off TestNG injection for a parameter. |
| **ObjectFactory** | Marks a method as the object factory to use for creating all test instances. |
| **Optional** | Specifies that the current parameter is optional. |
| **Parameters** | Describes how to pass parameters to a @Test method. |
| **Test** | Mark a class or a method as part of the test. |
| **TestInstance** | If this annotation is used on a parameter of a data provider, that parameter is the instance of the test method which is going to be fed by this data provider. |

---


|  |  |  |
| --- | --- | --- |
| **Overview** \| **Package** \| Class \| **Tree** \| **Deprecated** \| **Index** \| **Help** | |  |
| **PREV PACKAGE**   **NEXT PACKAGE** | **FRAMES**    **NO FRAMES**     **All Classes** |  |


---
